# Irving S. Wright Award of Distinction Lecture

**DOI:** 10.1093/geroni/igaa057.3197

**Published:** 2020-12-16

**Authors:** Nir Barzilai, Stephanie Lederman

## Abstract

The Irving S. Wright Award of Distinction Lecture will feature an address by the 2020 recipient James L. Kirkland, MD, PhD. This award is given by the American Federation for Aging Research, Inc.

